# Exploring the role of psychological well-being in the impact of hearing loss on depressive symptoms in rural older adults

**DOI:** 10.3389/fpubh.2025.1545483

**Published:** 2025-06-02

**Authors:** Qi Sun, Zhaoquan Jiang, Zhaoxu Xu, Mingyue Zhou, Xiaoyan Zhang, Tao Liu, Shixue Zhou

**Affiliations:** ^1^The First Hospital of Jinzhou Medical University, Jinzhou, Liaoning, China; ^2^School of Nursing, Jinzhou Medical University, Jinzhou, Liaoning, China; ^3^Department of Editorial (Social Sciences Edition), Jinzhou Medical University, Jinzhou, China

**Keywords:** rural older adults, hearing loss, psychological well-being, mediation effect, depressive symptoms

## Abstract

**Objective:**

Exploring the Relationship between Hearing Loss, psychological well-being, and Depressive Symptoms in Rural older adults: Validating the Mediating Role of psychological well-being in the Impact of Hearing Loss on Depressive Symptoms to Provide a Theoretical Basis for Improving the Mental Health of Rural older adults.

**Methods:**

The study focuses on 5,273 rural older individuals aged 65 and above, using data from the Chinese Longitudinal Healthy Longevity Survey (CLHLS) database. Hearing loss was assessed through self-report. Psychological well-being was evaluated using the Current Situation Assessment and Personality Emotional Characterization from the database. Depressive symptoms were measured using the short form of the Center for Epidemiologic Studies Depression Scale (CESD-10). The analysis involved using SPSS 27.0 and PROCESS v4.1 to conduct correlation analysis, regression analysis, and mediation effect analysis.

**Results:**

The depressive symptoms score for rural older individuals was (13.02 ± 4.48), and the psychological well-being score was (19.13 ± 2.47). There was a negative correlation between depressive symptoms and psychological well-being (r = −0.123, *p* < 0.001), there was a negative correlation between depressive symptoms and hearing loss (r = −0.086, *p* < 0.001). A negative correlation was also observed between hearing loss and psychological well-being (r = −0.060, *p* < 0.001). Psychological well-being mediates the effect of hearing loss on depressive symptoms among rural older individuals.

**Conclusion:**

Hearing loss in rural older individuals exerts both direct effects on depressive symptoms and indirect effects through the mediating role of psychological well-being. Specifically, the impairment of auditory function not only exacerbates emotional distress, but also diminishes psychological adaptability, thereby creating a dual pathway for depression development.

## 1 Introduction

In recent years, the prevalence and disability rates of hearing loss among the older adults have been rising, leading to a significant disease burden. Data indicate that in 2019, approximately 1.57 billion people worldwide had hearing loss, accounting for 20% of the global population (1). Hearing loss not only reduces quality of life but also contributes to the development of diseases such as dementia (2). Depression is one of the most common mental disorders among the older adults, with a prevalence of 20.0% among older adults in China (3). Depression is a leading cause of disability in the older adults, severely impacting their physical and mental health and adding stress to family and social relationships, thereby increasing the socioeconomic burden (4). Consequently, preventing depression in older adults is of significant public health importance.

Current research indicates a significant association between hearing loss and depressive symptoms. Studies have found that hearing loss can lead to social isolation, communication difficulties, and reduced self-esteem, which in turn increases the risk of depressive symptoms (5, 6). For rural older populations, where living conditions, medical resources, and social support are relatively limited, the impact of hearing loss and depressive symptoms may be even more pronounced. Psychological well-being reflects an individual’s emotional stability and adaptability in the face of stress and challenges. Hearing loss not only affects depressive symptoms but also negatively impacts psychological well-being. The communication barriers and reduced social interactions caused by hearing loss can lead to anxiety, loneliness, and identity crises, which weaken psychological well-being (7, 8). Research suggests that individuals with hearing loss exhibit lower emotional stability and adaptability under stress, resulting in reduced psychological well-being (9). Good psychological well-being can help individuals better cope with life’s pressures and challenges, thereby reducing the risk of depressive symptoms (10). Studies show that individuals with higher levels of psychological well-being have a significantly lower incidence of depressive symptoms compared to those with lower levels of psychological well-being (11).

Emerging evidence highlights that the relationship between hearing loss and depressive symptoms in older adults extends beyond psychosocial pathways to involve shared pathophysiological mechanisms. Neurobiologically, age-related hearing loss may accelerate cortical atrophy in brain regions critical for emotional regulation, impairing top-down control over negative affective states and heightening vulnerability to depression (12, 13). Additionally, chronic auditory deprivation disrupts neuroplasticity, potentially altering dopaminergic and serotonergic pathways implicated in mood disorders (14).

Peripherally, hearing loss is associated with systemic inflammation, marked by elevated pro-inflammatory cytokines, which are independently linked to depressive symptomatology (15). Furthermore, the increased cognitive load required to compensate for auditory deficits may overburden neural resources, exacerbating stress responses via hypothalamic–pituitary–adrenal (HPA) axis dysregulation and chronic cortisol elevation—a known risk factor for depression (16). Vascular comorbidities, common in aging populations, may also mediate this relationship by contributing to both auditory dysfunction and cerebrovascular damage in mood-regulating circuits (17, 18). These intertwined mechanisms suggest that hearing loss and depressive symptoms may mutually reinforce one another through bidirectional neurophysiological disruptions.

Based on existing research, this study hypothesizes that diminished psychological well-being (i.e., low mental health) mediates the effect of hearing loss on depressive symptoms. Specifically, hearing loss may increase the risk of depressive symptoms indirectly by reducing psychological well-being, rather than merely correlating with mental health as a broad construct. Using data from the Chinese Longitudinal Healthy Longevity Survey (CLHLS), this study explores the relationships among hearing loss, psychological well-being, and depressive symptoms in rural older individuals. The aim is to verify the mediating role of low psychological well-being in the pathway linking hearing loss to depressive symptoms, providing a scientific basis for developing targeted mental health interventions.

## 2 Methods

### 2.1 Study participants

The data used in this study were sourced from the 8th wave of the CLHLS project conducted by Peking University. The CLHLS is one of the most extensive and longest-running social science surveys in China, spanning from 1998 to 2018 and covering 23 provinces, municipalities, and autonomous regions. The cumulative sample size reached 113,000 participants. The 8th wave was conducted from 2017 to 2018 and included 15,874 individuals. The inclusion criteria for this study were as follows: (1) rural older individuals aged 65 and above; (2) availability of results from the Center for Epidemiologic Studies Depression Scale (CES-D-10); (3) availability of psychological well-being data; and (4) availability of hearing loss data. A total of 8,780 non-rural older individuals, 619 individuals without responses for depressive symptoms, and 1,202 individuals without responses for psychological well-being were excluded. A total of 5,273 rural older individuals met the inclusion criteria and were selected as the study population.

### 2.2 Research tools

1. Assessment of depressive symptoms: in this study, depressive symptoms were screened using the short version of the Center for Epidemiologic Studies Depression Scale (CES-D-10). This scale includes 10 items designed to assess depressive symptoms experienced by older individuals over the past week. Each item is scored from 0 to 3, with 2 items requiring reverse scoring. The total score ranges from 0 to 30, with a score of ≥10 indicating the presence of depressive symptoms and a score of <10 indicating their absence. The CES-D-10 has been widely used for screening depressive symptoms in older populations in China, demonstrating good reliability and validity with a Cronbach’s alpha coefficient of 0.78 (19, 20).
2. Hearing loss in this study was assessed through self-reported information from participants, a method validated in previous research (21, 22). Participants were first asked, “Do you have a disability involving deafness or partial deafness?” with responses coded as 0 (no impairment) or 1 (presence of impairment). To enhance assessment validity, we supplemented this measure with data on hearing aid usage by asking: “Do you currently use any hearing assistance devices (e.g., hearing aids or cochlear implants)?” Participants reporting hearing aid use were analyzed separately as a subgroup and included as a covariate in sensitivity analyses to account for potential mitigation of hearing impairment effects.
3. Assessment of psychological well-being: based on established indicators and calculation methods from previous studies (23–26), we developed the assessment for psychological well-being. This study evaluated psychological well-being through questions in the “Current Situation Assessment and Personality Emotional Traits” questionnaire, which captures older individuals’ subjective views on their overall quality of life and living standards, thereby providing a measure of psychological well-being. The survey included three questions reflecting positive emotions: “How do you feel about your life right now?,” “Can you think about what’s happening?,” and “Do you feel energetic?” Negative emotions were assessed with three questions: “Do you feel ashamed, regretful, or guilty about things you have done?,” “Do you feel angry at people or things you dislike?,” and “Do you often feel people around you are untrustworthy?” Responses to the positive emotion questions were reverse scored, from “very good” (5 points) to “very poor” (1 point). In contrast, the negative emotion questions were scored directly, with “always” scoring 1 point and “never” scoring 5 points. This standardization facilitated data measurement and calculation. Scores for positive and negative emotions each ranged from 3 to 15, with an overall psychological well-being score ranging from 6 to 30. Higher scores indicate better psychological well-being. In this research, the scale showed strong internal consistency, with a Cronbach’s *α* value of 0.860.

### 2.3 Data analysis

Data were analyzed using SPSS version 27.0. Quantitative data were expressed as mean ± standard deviation, while qualitative data were reported as frequency and composition ratio. Pearson correlation analysis was conducted to examine the relationships among hearing loss, psychological well-being, and depressive symptoms in rural older individuals. The mediation effect of psychological well-being between hearing loss and depressive symptoms was analyzed using the Process 4.1 plugin, with the Bootstrap method applied to test the mediation effect through 5,000 resampling iterations to calculate the 95% confidence interval. A *p* < 0.05 was considered statistically significant.

## 3 Results

### 3.1 Demographic information of rural older adults

The results of this study show that the age range of participants was 67–117 years, with an average age of 82.8 ± 11.1 years. Gender distribution included 2,343 males (46.1%) and 2,930 females (53.9%). Ethnicity distribution was as follows: 3,992 Han Chinese (75.7%) and 1,281 individuals from other ethnic groups (24.3%). Educational levels were categorized as follows: primary school or below for 1,859 individuals (35.3%), middle school for 972 individuals (18.4%), and high school or above for 2,442 individuals (46.3%). Marital status showed that 2,912 participants (55.2%) were without a spouse, while 2,361 participants (44.8%) had a spouse. Additionally, 929 participants (17.6%) were smokers, and 856 participants (16.2%) consumed alcohol.

### 3.2 Psychological well-being and depressive symptom scores and the relationship between hearing impairment, psychological well-being and depressive symptoms in rural older adults

The depressive symptom score among rural older individuals was 13.02 ± 4.48, and the psychological well-being score was 19.13 ± 2.47. There was a negative correlation between depressive symptom scores and psychological well-being (r = −0.123, *p* < 0.001) and a negative correlation between depressive symptom scores and hearing loss (r = −0.086, *p* < 0.001). Additionally, there was a negative correlation between hearing loss and psychological well-being (r = −0.060, *p* < 0.001) (Table 1).

**Table 1:** Analysis of the mediating effect of psychological well-being among rural older adults between the effects of hearing impairment on depressive symptoms.

Regression equation	Predictor variable	Overall fit index			Significance	
Outcome variable		R	R^2^	F	B (95%CI)	*t*
Psychological well-being	Gender	0.074	0.006	4.344	−0.203* (−0.374–−0.031)	−2.314
	Ethnic group				0.026 (−0.048–0.099)	0.689
	Smoke				0.174 (−0.053–0.399)	1.505
	Drink				0.167 (−0.062–0.396)	1.430
	Hearing impairment				−0.310* (−0.474–−0.146)	−0.317
Depressive symptoms	Gender	0.197	0.039	24.483	0.611* (0.310–0.912)	3.974
	Ethnic group				0.189* (0.059–0.318)	2.863
	Smoke				−0.120 (−0.517–0.277)	−0.591
	Drink				0.841* (0.438–1.243)	4.096
	Hearing impairment				−0.965* (−1.253–−0.676)	−6.558
	Psychological well-being				−0.222* (−0.277–−0.167)	−7.949
Depressive symptoms	Hearing impairment	0.153	0.023	18.846	−0.896* (1.186–−0.606)	−6.054
	Gender				0.656* (0.352–0.960)	4.236
	Ethnic group				0.183* (0.053–0.313)	2.755
	Smoke				−0.158* (−0.558–0.242)	−0.775
	Drink				0.803* (0.398–1.209)	3.886

*p < 0.01. Control gender, ethnic group, smoke, drink.

### 3.3 Analysis of the mediating effect of psychological well-being between hearing impairment and depressive symptoms

**Figure 1:**
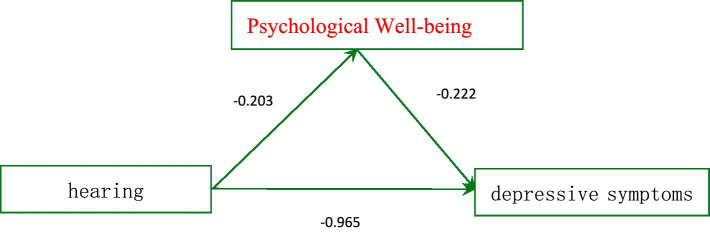
Mediating effect of psychological well-being in the effect of hearing impairment on depressive symptoms.

A mediation model was constructed with hearing loss as the predictor variable, depressive symptoms as the outcome variable, and psychological well-being as the mediating variable. Mediation analysis was conducted using Model 4 of the SPSS Process component. The results indicated that hearing loss had a significant negative predictive effect on depressive symptoms (*β* = −0.965, *p* < 0.001), while psychological well-being had a significant negative predictive effect on depressive symptoms (*β* = −0.222, *p* < 0.001). Additionally, hearing loss significantly negatively predicted psychological well-being (*β* = −0.203, *p* < 0.001). These results are shown in Table 2, and the path coefficients among the variables are illustrated in Figure 1.

**Table 2:** Direct and mediating effects.

Hearing impairment → depressive symptoms	Effect value	BootSE	95% percentile confidence interval	
			Lower limit	Upper limit
Total effect	−0.920	0.148	−0.606	−1.186
Direct effect	−0.965	0.147	−1.253	−0.676
Indirect effect	0.045	0.021	0.031	0.114

To further examine the mediation effect, a bias-corrected nonparametric percentile Bootstrap method, as provided by Hayes, was applied with 5,000 resamples and a 95% confidence interval. The results (see Table 2) revealed a significant indirect effect of hearing loss on depressive symptoms through psychological well-being (mediation effect = 0.045, SE = 0.021), with a 95% confidence interval [0.031, 0.114] that did not include zero, indicating that psychological well-being mediated the relationship between hearing loss and depressive symptoms among rural older individuals. The mediation effect accounted for 4.9% of the total effect.

## 4 Discussion

The results of this study indicate a negative correlation between hearing loss and depressive symptoms among rural older individuals (r = −0.086, *p* < 0.001), suggesting that more severe hearing loss may be associated with higher scores of depressive symptoms. The impact of hearing loss on depressive symptoms in the older adults is complex; in addition to increased social isolation and psychological burden, hearing loss may also lead to declining physical health, increased risk of chronic diseases, and poorer sleep quality—all physiological factors linked to depression (13, 27).

A negative correlation was found between hearing loss and psychological well-being (r = −0.060, *p* < 0.001), indicating that worsening hearing loss may affect psychological well-being in older individuals. Due to the cognitive load required to cope with hearing difficulties, psychological well-being may become compromised (28). Hearing loss can diminish self-efficacy, impacting confidence and ability to face challenges, thereby lowering psychological well-being levels (29). Additionally, psychological well-being scores among rural older individuals were negatively correlated with depressive symptoms (r = −0.123, *p* < 0.001), meaning that lower psychological well-being scores were associated with more severe depressive symptoms. This negative correlation reflects the potential influence of psychological well-being on depressive symptoms. In rural older populations, psychological well-being encompasses not only emotional stability but also adaptability to life’s challenges and self-regulation mechanisms. Studies indicate that poor psychological well-being may hinder effective emotional regulation, thereby increasing the risk of depressive symptoms (30). Furthermore, with aging, cognitive function in the older adults gradually declines, particularly affecting cognitive flexibility and decision-making ability, which increases susceptibility to depression. This finding is supported by studies, such as those by Kuo et al. (31).

Psychological well-being plays an important role in mitigating the impact of hearing loss on depressive symptoms among rural older individuals. Firstly, it enhances emotional regulation, helping individuals cope more effectively with the negative emotional responses caused by hearing loss. This includes cognitive regulation of emotions and emotional expression, enabling older individuals to face various life challenges and stresses more resiliently (32–36). Additionally, good psychological well-being helps protect mental resources, including cognitive abilities, emotional support, and coping strategies, reducing the potential depletion of these resources due to hearing loss. This protective effect helps maintain a more stable emotional state and higher life satisfaction among older individuals (37).

Furthermore, psychological well-being strengthens social support networks (such as family, friends, and community), providing emotional security and tangible support. This support and connection not only help older individuals better cope with the psychological stress and loneliness associated with hearing loss but also reduce the occurrence of depressive symptoms (38).

The analysis above demonstrates that psychological well-being plays a crucial mediating role in alleviating the impact of hearing loss on depressive symptoms among rural older individuals. A deeper understanding of these mechanisms and pathways can aid in developing targeted interventions to improve mental health and quality of life for the older adults. Based on this, the following measures are proposed to reduce depressive symptoms in rural older populations:

Prioritize personalized hearing aid fitting, auditory rehabilitation training, and regular hearing assessments to directly mitigate neurobiological stress responses caused by sensory deprivation. Implement cognitive-behavioral therapy (twice weekly) and digital emotion regulation training alongside auditory rehabilitation. Use virtual reality (VR) to simulate social scenarios for auditory-emotional integration exercises.

Establish a “Hearing-Friendly Community” certification program, mandating loop amplification systems in public spaces (39). Develop dialect-adaptive hearing aids and deploy interdisciplinary teams (audiologists + rural social workers + AI technicians) for monthly in-home device maintenance and psychological evaluation (40).

Design dual-task protocols combining auditory discrimination and cognitive exercises, enhanced by transcranial direct current stimulation (tDCS) to boost auditory cortex plasticity. Implement EEG biofeedback systems for real-time emotion regulation, dynamically adjusting intervention intensity based on neural activity.

Launch an intergenerational communication app that automatically optimizes grandchildren’s speech for high-frequency enhancement. Create a “Sound Memory Museum” digital platform using reminiscence therapy to rebuild auditory self-efficacy.

Apply machine learning to predict hearing loss trajectories, initiating 6-month preemptive cognitive reserve enhancement for high-risk individuals. Establish a rural auditory biobank to investigate gene–environment interactions affecting depression susceptibility.

## 5 Limitation

While this study provides valuable insights into the relationship between hearing loss, psychological well-being, and depressive symptoms in rural older individuals, several limitations should be acknowledged.

A noteworthy consideration is the potential role of suppression effects in the relationship between hearing loss, psychological well-being, and depressive symptoms. While our findings highlight psychological well-being as a mediator that amplifies the association between hearing loss and depressive symptoms (i.e., hearing loss reduces psychological well-being, which in turn exacerbates depressive symptoms), suppression effects may arise if unaccounted variables indirectly oppose the total effect. For instance, certain factors (e.g., compensatory social engagement or adaptive coping strategies triggered by hearing loss) could theoretically counteract the negative psychological consequences, creating a suppression mechanism where the indirect effect opposes the direct or total effect (41). Although our current model did not identify such suppression pathways, this possibility underscores the complexity of psychosocial mechanisms in aging populations. Future studies should explicitly test for suppression effects using moderated mediation or countervailing pathway analyses to disentangle these dynamics.

The data used in this study are cross-sectional, meaning that only a snapshot of the participants’ conditions was captured at a single point in time. As a result, the causality between hearing loss, psychological well-being, and depressive symptoms cannot be definitively established. Longitudinal studies would be beneficial in providing a clearer understanding of the temporal relationships between these variables and in identifying causal pathways.

Hearing loss in this study was assessed based on self-reported data, which may be subject to recall bias or misclassification. Participants’ perceptions of their hearing abilities may not fully reflect the actual severity of their hearing loss, as they might not accurately recognize or report the extent of their hearing difficulties. Future studies should consider objective hearing assessments (e.g., audiometric testing) to validate self-reported hearing loss and obtain more accurate data.

The assessment of psychological well-being was based on a questionnaire that captured subjective views on quality of life and emotional traits. While this tool demonstrated strong internal consistency, it remains a self-report measure, which can be influenced by individual perceptions, mood states, or social desirability bias. Additionally, the scale might not capture all dimensions of psychological well-being, and future research could benefit from incorporating more comprehensive measures that assess additional factors such as life satisfaction, social connectedness, and coping strategies.

This study focused specifically on rural older individuals, and the findings may not be generalizable to urban populations or to older individuals in other geographic regions of China or internationally. Rural populations often face unique social, economic, and healthcare challenges that may not be reflective of those encountered by urban older populations. Thus, caution is needed when interpreting these results in a broader context.

Although this study accounted for key variables such as depressive symptoms and hearing loss, there may be other potential confounders that were not included in the analysis. Factors such as socioeconomic status, social support, chronic illnesses, and access to healthcare could also play a significant role in the relationship between hearing loss, psychological well-being, and depressive symptoms. Future research should consider including these variables to further refine our understanding of the interplay between these factors.

While the study sample of 5,273 rural older individuals is substantial, the inclusion criteria focused specifically on older individuals with available data for depressive symptoms, psychological well-being, and hearing loss. As a result, there may be selection bias in the sample, potentially excluding individuals who may be more vulnerable to depressive symptoms but who were not included due to missing data or other reasons. Efforts to address missing data and ensure a more representative sample in future studies could enhance the generalizability of the findings.

Despite these limitations, this study provides important insights into the role of psychological well-being in the relationship between hearing loss and depressive symptoms among rural older individuals, suggesting potential avenues for future research and interventions aimed at improving the mental health of this population.

## Data Availability

The datasets presented in this study can be found in online repositories. The names of the repository/repositories and accession number(s) can be found in the article/supplementary material.
